# Association Between Thrombus Density and Reperfusion Outcomes Using Different Thrombectomy Strategies: A Single-Center Study and Meta-Analysis

**DOI:** 10.3389/fneur.2019.00843

**Published:** 2019-08-16

**Authors:** Gengfan Ye, Ruoyao Cao, Jun Lu, Peng Qi, Juan Chen, Daming Wang

**Affiliations:** ^1^Department of Neurosurgery, Beijing Hospital, National Center of Gerontology, Beijing, China; ^2^Graduate School of Peking Union Medical College, Beijing, China; ^3^Beijing Institute of Geriatrics, Beijing Hospital, National Center of Gerontology, Beijing, China; ^4^Department of Radiology, Beijing Hospital, National Center of Gerontology, Beijing, China

**Keywords:** acute ischemic stroke, thrombus density, CT, reperfusion, thrombectomy

## Abstract

**Background:** For patients with acute ischemic stroke (AIS), the thrombus density on non-enhanced CT (NECT) indicates the composition of the thrombus, a characteristic that impacts the efficacy of mechanical thrombectomy (MT). A previous meta-analysis suggested a correlation between higher thrombus density and successful reperfusion, but some new studies have drawn different conclusions. This single-center study and meta-analysis aimed to detect the association between thrombus density and reperfusion outcomes based on various thrombectomy strategies.

**Methods:** We reviewed AIS patients who underwent MT at our center between July 2015 and May 2019. Thrombus density was recorded as mean Hounsfield Unit (HU) value on 1-mm reconstructed NECT, and expanded Thrombolysis In Cerebral Infarction (eTICI) scale was used to evaluate the reperfusion grade. The difference in thrombus density was examined according to reperfusion outcomes. Then, we systematically searched relevant literature on this issue. The random effect model was used to calculate standardized mean difference (SMD), and subgroup analysis was conducted according to MT strategies employed, including stent retriever (SR), contact aspiration (CA), Solumbra (a combination of SR and aspiration), and multiple thrombectomy modalities.

**Results:** Sixty-four patients with anterior circulation AIS were included in our single-center study with 57 (89.1%) achieving successful reperfusion (eTICI2b-3). Retrospective analysis showed no significant difference in thrombus density between eTICI2b-3 and eTICI0-2a reperfusion (65.27 vs. 62.19, *p* = 0.462). As for systematic review, 11 studies were included in qualitative analysis, among which 6 had data available for meta-analysis. Pooled result showed that a comparable thrombus density between eTICI2b-3 and eTICI0-2a reperfusion (SMD 0.14, 95%CI −0.28 to 0.57, *p* = 0.50). Interestingly, in the SR subgroup, eTICI2b-3 reperfusion showed a significant higher thrombus density (SMD 0.53, 95%CI 0.10 to 0.96, *p* = 0.02), while an inverse trend was observed in the CA subgroup (SMD −0.48, 95%CI −0.88 to −0.07, *p* = 0.02).

**Conclusions:** Although the pooled result of meta-analysis did not show a significant association between thrombus density and successful reperfusion, subgroup analysis implicated that the SR technique might be prone to retrieve high-density thrombus, while the CA subgroup showed an opposite tendency. Further studies are needed to confirm these results and to investigate its role in the optimization of thrombectomy strategy.

## Introduction

The treatment for acute ischemic stroke (AIS) has advanced rapidly and mechanical thrombectomy (MT) has been proven to be superior to intravenous thrombolysis for patients with intracranial large vessel occlusion (1–6). Though successful reperfusion (expanded Thrombolysis In Cerebral Infarction (eTICI) 2b-3) could be achieved in most cases, there is still part of thrombi reluctant to remove. This might be partially attributed to the histopathologic nature of thrombus: despite the controversy, several studies indicated that red blood cell (RBC)-rich clot was more responsive to MT, while fibrin-rich thrombus was more refractory (7–9).

Although histologic information of thrombus is not available before thrombectomy, it seems that the Hounsfield Unit (HU) value on non-enhanced CT (NECT) scanning could implicate the composition of thrombi to some extent (9–11). A systematic review suggested that the hyperdense thrombus indicated a high RBC content (10). Maekawa et al. also found that the CT attenuation of RBC-rich clot was significantly higher than that of fibrin-rich clot (9). Moreover, Sporns et al. indicated that the relative HU value of thrombus had a significant positive correlation with its RBC content (11). Given the association between thrombus density and histologic feature, the HU value might be an important tool for us to know about the composition of clot before thrombectomy.

Several studies have reported the correlation between thrombus density on NECT and reperfusion outcomes of thrombolysis and thrombectomy treatment. Additionally, Brinjikji et al. performed a meta-analysis on this issue finding a significant association between higher thrombus density and successful reperfusion (10). However, the results of thrombolysis and thrombectomy were pooled together in that meta-analysis, which was inappropriate. Furthermore, some newer studies have drawn different conclusions. As a consequence, we aimed to update the systematic review and meta-analysis on the correlation between thrombus density and reperfusion outcome of MT, and to perform subgroup analysis for exploring the role of different thrombectomy strategies, especially stent retriever (SR), and contact aspiration (CA). In addition, we carried out a retrospective observational study based on our single center experience and the results would also be added into the meta-analysis.

## Materials and Methods

### Methods for Single Center Study

#### Patient Selection

This was a retrospective observational study with most clinical data collected prospectively. We consecutively reviewed medical records of AIS patients who underwent endovascular treatments (EVTs) at our center (Beijing Hospital, National Center of Gerontology) from July 2015 to May 2019. All patients suspected suffering from AIS underwent NECT, and most underwent additional multimodal CT (including NECT, CT angiography, CT perfusion). There was no specific restriction on the National Institutes of Health Stroke Scale (NIHSS), Alberta Stroke Programme Early CT Score (ASPECTS), and duration from onset to treatment. EVT indication was evaluated by the attending neurointerventionalist (JL or PQ).

Inclusion/exclusion criteria were as follows: (1) Only anterior circulation AIS patients were included; (2) Patients who underwent thrombectomy were included, including SR, CA, and Solumbra (a combination of SR and aspiration); (3) Those patients who received only intra-arterial thrombolysis (IAT) or percutaneous transluminal angioplasty and/or stenting (PTA/S) were excluded; (4) Data on 1-mm thickness reconstructed NECT was available; and (5) Patients were excluded if the thrombectomy devices could not reach the thrombus location.

#### Clinical Data Collection

The following baseline characteristics were recorded: demographic features (age and sex), comorbidities (including atrial fibrillation, hypertension, diabetes mellitus, dyslipidemia, coronary artery disease, stroke or transient ischemic attacks (TIA) history, smoking history), whether received intravenous thrombolysis (IVT), and stroke etiology. Stroke etiology was categorized according to Trial of Org 10,172 in Acute Stroke Treatment (TOAST) classification (12).

The thrombus location, primary treatment strategy and reperfusion outcomes were collected according to procedure notes. The thrombus location was classified as the intracranial internal carotid artery (ICA), the first segment of middle cerebral artery (M1), tandem lesion, and distal part. The distal artery was defined as the second segment of middle cerebral artery (M2) or anterior cerebral artery (A1/A2). The primary treatment strategies were categorized as SR, CA, and Solumbra. The reperfusion grade was evaluated according to the eTICI scale (13). The successful and complete reperfusion was defined as eTICI2b-3 and eTICI2c-3, respectively; the reperfusion outcomes after first pass, after primary strategy, and final outcomes were recorded, respectively.

#### Imaging Protocol and Thrombus Density Measurement

Images were acquired on a 320 ×0.5 mm detector rows CT scanner (Aquilion ONE, Canon Medical Systems). The parameters for NECT were as follows: 80 kV, 200 mAs, and 5-mm reconstructed slice thickness; while the multimodal scanning parameters were: 80 kV, 100 mAs, and 1-mm reconstructed slice thickness.

Two trained raters (GFY and RYC) measured the thrombus density for each case independently. First, the thrombus location was determined by contrast-enhanced CT (CECT). Then, a round region of interest (ROI) accounting for about two-thirds of the vascular area was drawn and placed within the thrombus ~2 mm behind the occlusion site. The mean HU value on 1-mm NECT was recorded as thrombus density. If the measurements showed an obvious difference (>10 HU) between two raters, the disagreement was resolved by consensus or with the help of the senior investigator (JC). Interclass correlation coefficient (ICC) was calculated to evaluate the inter-rater reliability. The ICC for the HU value of thrombus was 0.899, which showed a high inter-rater agreement.

#### Statistical Analysis

Patients included were assigned to three groups according to primary strategies (SR, CA, or Solumbra), and the differences in baseline characteristics among these groups were examined. Normality testing was used for continuous variable (age and thrombus density). As age was inconsistent with normal distribution, it was expressed as median (interquartile range, IQR) and Kruskal–Wallis test was used to detect the difference among groups. The HU value conformed to normal distribution, so the mean (standard deviation, SD) and analysis of variance (ANOVA) were adopted. As for the categorical variable, count (percent) and R × C chi-square test were used. Then, we compared the thrombus density according to the different reperfusion outcomes (eTICI0-2a/eTICI2b-3 and eTICI0-2b/eTICI2c-3) using two-independent sample *t*-test. Three different conditions were considered including reperfusion after first-pass, after primary strategy, and at the end of procedure. Subgroup analysis was conducted according to primary strategies (SR, CA, Solumbra).

All tests used α-level of 0.05 for significance. These statistical analyses were performed with SPSS software version 23.0 and the forest plot of subgroup analysis was drawn with R software version 3.5.0.

### Methods for Meta-Analysis

#### Protocol and Guidance

The study was conducted in adherence with the Preferred Reporting Items for Systematic Reviews and Meta-Analyses (PRISMA) Statement and was registered at the International Prospective Register of Systematic Reviews (CRD42019120841) (14).

#### Search Strategy and Eligibility Criteria

The literature published before June 5, 2019 was systematically searched on PubMed, Embase, and Cochrane databases with no language restriction. The keyword for the population was “stroke.” “HU,” “rHU,” or “hyperdens^*^” were used as keywords for exposure. Meanwhile, “recanalization,” “revascularization,” or “reperfusion” were used as the keywords for the outcome.

Two investigators (GFY and RYC) independently reviewed each entry. Disagreements were resolved by consensus or with the help of the senior investigator (DMW). Those studies were regarded eligible if they reported the correlation between thrombus density and reperfusion outcomes. The total sample size should be >10. For those studies with duplicate cases, only the complete reports were included. Case reports and reviews were excluded.

#### Data Extraction and Risk of Bias Assessment

Two investigators (GFY and RYC) independently extracted all baseline data and main outcomes from each eligible study: demographic profiles (total number of patients, age, and gender), thickness of CT scan, method of region of interest (ROI) drawing, baseline NIHSS, whether received IVT before MT, stroke etiology, lesion location, thrombectomy modalities and devices, and the HU value of thrombus. Besides, the statistical methods and main conclusions were also collected from those studies included for qualitative analysis. Disagreements were resolved by consensus or with the help of the senior investigator (JC).

To evaluate the risk of bias, two investigators (GFY and RYC) independently assessed each article according to the modified Newcastle–Ottawa Scale (mNOS), which is designed to assess the quality of non-randomized studies included in systematic reviews and meta-analysis (15). Each study was assessed based on three aspects: selection of cases, comparability of cases, and ascertainment of exposure. Disagreements were resolved by consensus or with the help of the senior investigator (JC).

#### Statistical Analysis

Two investigators (GFY and RYC) independently analyzed the data and performed the synthesis. Disagreements were resolved by consensus or with the help of the senior investigator (DMW). The standardized mean difference (SMD) in thrombus density between eTICI2b-3 and eTICI0-2a reperfusion was calculated in the meta-analysis. The random effects model was used to pool these outcomes (16). The final reperfusion outcomes of our single-center study was included in the pooled results of meta-analysis.

Publication bias was evaluated qualitatively by the asymmetry of the funnel plot and quantitatively by the Egger test, which is defined as significant when *p* < 0.1. Heterogeneity among studies was assessed by calculating the Q-statistic and the value of *I*^2^. It was considered statistically significant when the *p* value of Q-statistic < 0.05; the value of *I*^2^ was used to estimate the magnitude of heterogeneity when *I*^2^ > 50% indicated a moderate-to-high heterogeneity (17). Sensitivity and subgroup analysis were used to detect the sources of heterogeneity. Subgroup analysis was conducted according to treatment strategies (including SR, CA, Solumbra, and multiple treatment modalities). The reperfusion outcomes after primary strategies of our single-center study were included in the subgroup analysis of the meta-analysis.

These statistical analyses were conducted using Review Manager version 5.3 and Stata version 12.0.

## Results

### Results of the Single-Center Study

#### Patients Selection and Baseline Characteristics

A total of 114 AIS patients underwent endovascular treatments at our center between July 2015 and May 2019. Twenty-five patients were excluded due to posterior circulation stroke and 17 patients were excluded because the 1-mm-thickness NECT data were unavailable. In addition, four patients who only received IAT were excluded, three patients who underwent primary PTA/S were excluded, and another patient was excluded for failure to pass the carotid tandem lesion.

A total of 64 patients (median age, 74.5 years; 34 male) were included in the final analysis and their overall baseline characteristics are shown in Table 1. Among these patients, 37 (57.8%) were diagnosed with cardiogenic embolism, 16 (25.0%) with large artery atherosclerosis, 2 (3.1%) with other causes, and 9 (14.1%) had cryptogenic stroke. As for thrombus location, 18 (28.1%) were in ICA, 25 (39.1%) were in M1, 14 (21.9%) were in the distal part, and 7 (10.9%) were tandem lesions. Nineteen patients (29.7%) received IVT before thrombectomy and the mean thrombus density on 1-mm NECT was 64.94 (SD, 10.36).

**Table 1:** Baseline characteristics.

	**Total (*n* = 64)**	**SR (*n* = 21)**	**CA (*n* = 26)**	**Solumbra (*n* = 17)**	***P*-value**
**Demographics**					
Age, year (median, IQR)	74.5 (63.25–82)	70 (61.5–79.5)	78.5 (65.75–84)	78 (66–83.5)	0.286
Sex, male (*n*, %)	34 (53.1%)	13 (61.9%)	13 (50.0%)	8 (47.1%)	0.606
**Comorbidities (*****n*****, %)**					
Atrial fibrillation	32 (50.0%)	8 (38.1%)	16 (61.5%)	8 (47.1%)	0.268
Hypertension	47 (73.4%)	16 (76.2%)	20 (76.9%)	11 (64.7%)	0.635
Diabetes mellitus	25 (37.9%)	12 (57.1%)	7 (26.9%)	4 (23.5%)	0.046
Dyslipidemia	27 (42.2%)	10 (47.6%)	9 (34.6%)	8 (47.1%)	0.597
Stroke or TIA history	21 (32.8%)	6 (28.6%)	12 (46.2%)	3 (17.6%)	0.132
Smoking history	22 (34.4%)	11 (52.4%)	7 (26.9%)	4 (23.5%)	0.103
Coronary artery disease	25 (39.1%)	8 (38.1%)	12 (46.2%)	5 (29.4%)	0.543
Preoperative IVT (*n*, %)	19 (29.7%)	6 (28.6%)	6 (23.1%)	7 (41.2%)	0.442
**Stroke etiology (*****n*****, %)**					0.115
CE	37 (57.8%)	8 (38.1%)	19 (73.1%)	10 (58.8%)	
LAA	16 (25.0%)	6 (28.6%)	6 (23.1%)	4 (23.5%)	
Other determined	2 (3.1%)	1 (4.8%)	0 (0%)	1 (5.9%)	
Cryptogenic	9 (14.1%)	6 (28.6%)	1 (3.8%)	2 (11.8%)	
**Thrombus location (*****n*****, %)**					0.003
ICA	18 (28.1%)	4 (19.0%)	10 (38.5%)	4 (23.5%)	
M1	25 (39.1%)	6 (28.6%)	9 (34.6%)	10 (58.8%)	
Tandem	7 (10.9%)	0 (0%)	5 (19.2%)	2 (11.8%)	
Distal	14 (21.9%)	11 (52.4%)	2 (7.7%)	1 (5.9%)	
Thrombus density on 1-mm NECT (mean HU, SD)	64.94 ± 10.36	62.59 ± 12.35	64.90 ± 9.95	67.89 ± 7.80	0.296

*Date are shown as mean ± SD, median (IQR) or number (percent)*.

*TIA, transient ischemic attacks; IVT, intravenous thrombolysis; CE, cardiogenic embolism; LAA, large artery atherosclerosis; SR, stent retriever; CA, contact aspiration; Solumbra, a combination of SR and aspiration; NECT, non-enhanced computed tomography; HU, Hounsfield Unit*.

All 64 patients were assigned to three groups according to primary thrombectomy strategy: SR (21, 32.8%), CA (26, 40.6%), and Solumbra (17, 26.6%).The comparisons among these three groups are shown in Table 1; only the proportion of diabetes mellitus and thrombus location showed significant differences. There was no significant difference observed for the other baseline characteristics.

#### Correlation Between HU Value and Reperfusion Outcomes

The comparisons of HU value according to the different reperfusion outcomes are shown in Table 2. After the first pass of thrombectomy, 17 patients (26.6%) achieved eTICI2c-3 and no significant difference in thrombus density was observed between eTICI2c-3 and eTICI0-2b reperfusion (65.99 vs. 64.56, *p* = 0.630). Twenty patients (31.3%) obtained eTICI2b-3 after the first maneuver and there was also no significant difference observed between eTICI2b-3 and eTICI0-2a reperfusion (67.18 vs. 63.92, *p* = 0.246).

**Table 2:** Reperfusion outcomes of different primary MT strategies.

	**TICI 2c-3**		**TICI 0-2b**		***P*-value**	**TICI 2b-3**		**TICI 0-2a**		***P*-value**
	**HU**	***n***	**HU**	***n***		**HU**	***n***	**HU**	***n***	
First-pass outcome	65.99 ± 8.44	17	64.56 ± 11.03	47	0.630	67.18 ± 9.46	20	63.92 ± 10.70	44	0.246
Primary SR	71.39 ± 8.47	6	59.07 ± 12.08	15	0.035	69.64 ± 9.01	7	59.06 ± 12.54	14	0.062
Primary CA	57.45 ± 5.22	5	66.68 ± 10.06	21	0.061	60.44 ± 8.69	6	66.24 ± 10.12	20	0.217
Primary Solumbra	67.70 ± 4.80	6	68.00 ± 9.26	11	0.942	70.49 ± 8.59	7	66.07 ± 7.07	10	0.286
Primary strategy outcome	65.34 ± 9.97	29	64.61 ± 10.82	35	0.781	66.01 ± 10.22	34	63.72 ± 10.56	30	0.383
Primary SR	68.07 ± 11.10	9	58.50 ± 12.0	12	0.078	67.18 ± 10.84	10	58.42 ± 12.63	11	0.106
Primary CA	59.91 ± 10.68	10	68.02 ± 8.34	16	0.040	61.98 ± 10.88	12	67.41 ± 8.70	14	0.170
Primary Solumbra	68.31 ± 5.95	10	67.30 ± 10.41	7	0.802	69.07 ± 8.36	12	65.08 ± 6.11	5	0.353
Final outcome	64.76 ± 9.22	49	65.52 ± 13.84	15	0.805	65.27 ± 9.42	57	62.19 ± 17.01	7	0.462
Primary SR	64.38 ± 9.92	16	56.87 ± 18.45	5	0.245	64.07 ± 9.69	17	56.30 ± 21.26	4	0.269
Primary CA	63.36 ± 10.28	21	71.39 ± 5.10	5	0.106	64.65 ± 10.28	24	67.90 ± 5.30	2	0.667
Primary Solumbra	67.72 ± 5.56	12	68.31 ± 12.57	5	0.892	67.49 ± 7.87	16	74.35	1	NA

*Thrombus density are shown as mean HU ± SD*.

*SR, stent retriever; CA, contact aspiration; Solumbra, a combination of stent retriever and aspiration; HU, Hounsfield unit; NA, not available*.

For reperfusion outcome after primary strategy, 29 patients (45.3%) and 34 patients (53.1%) achieved eTICI2c-3 and eTICI2b-3, respectively. The HU value of thrombus showed no significant difference between eTICI2c-3 and eTICI0-2b reperfusion (65.34 vs. 64.61, *p* = 0.781), as well as between eTICI2b-3 and eTICI0-2a reperfusion (66.01 vs. 63.72, *p* = 0.383).

For final reperfusion outcome, 49 patients (76.6%) obtained eTICI2c-3 and 57 patients (89.1%) achieved eTICI2b-3. Also, there was no significant difference observed between eTICI2c-3 and eTICI0-2b reperfusion (64.76 vs. 65.52, *p* = 0.805), as well as between eTICI2b-3 and eTICI0-2a reperfusion (65.27 vs. 62.19, *p* = 0.462).

**Figure 1:**
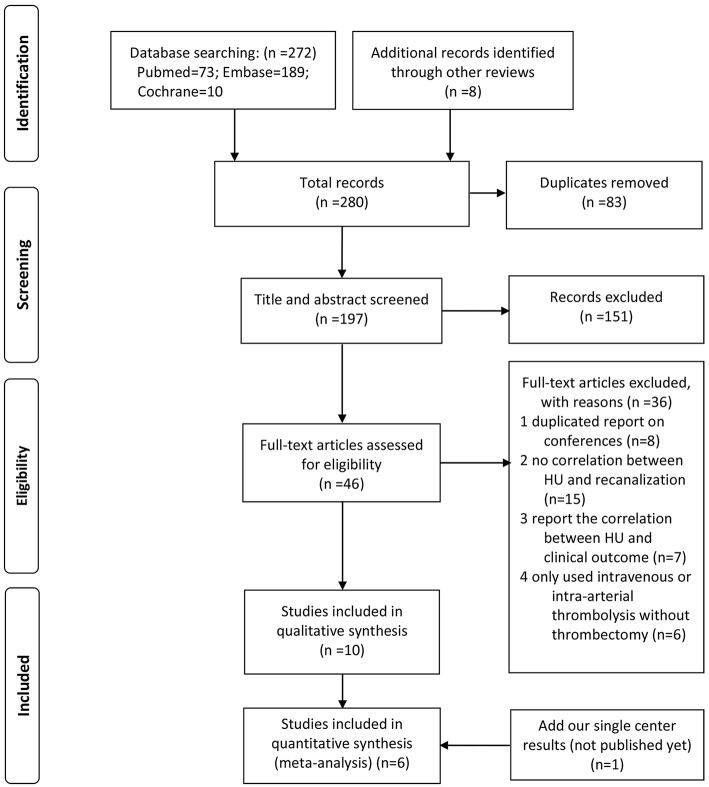
PRISMA flow diagram.

In order to assess the correlation between reperfusion outcomes and each treatment strategy, further subgroup analysis and forest plots were executed according to different primary thrombectomy strategy (Table 2 and Figure 1). For subgroups based on the primary SR technique, thrombus density in the eTICI2b-3 or eTICI2c-3 reperfusion group was relatively higher than that in the eTICI0-2a or eTICI0-2b reperfusion group. An inverse tendency was observed for the primary CA subgroup without significant difference.

### Results of Meta-Analysis

#### Studies Selection and Characteristics

**Figure 2:**
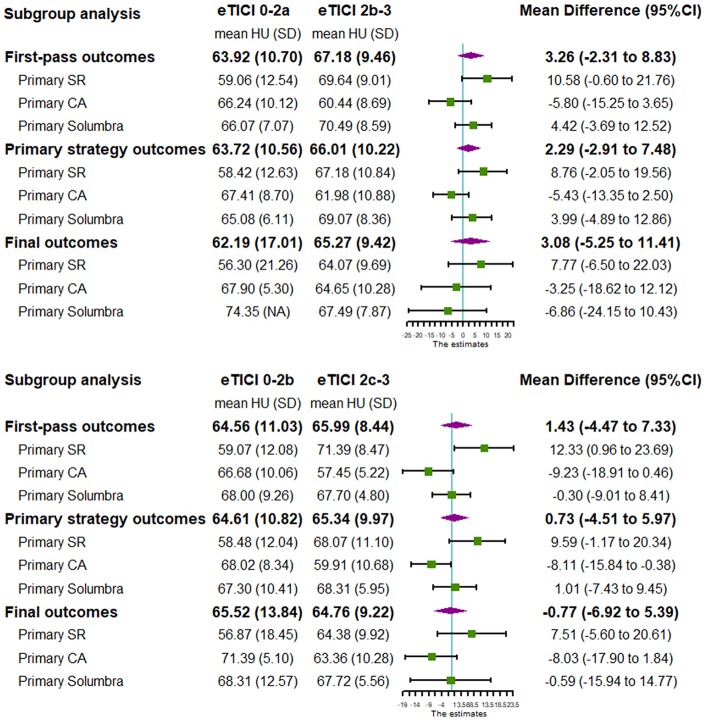
Forest plot shows subgroup analysis of our single center study.

As shown in the flow diagram (Figure 2), we identified 280 articles. Finally, 11 studies (including our single-center study) were included (18–27) (9 articles and 2 conference abstracts) for qualitative synthesis, among which 6 studies had data available for quantitative synthesis. All these six articles included in the meta-analysis had moderate-to-high quality according to the mNOS (Supplementary Table 1).

The characteristic of each study is shown in Table 3. A total of 439 AIS cases that underwent MT were included in the meta-analysis. The measurement of thrombus density was on 5-mm-thickness NECT in most studies, while Shu et al. (21) used 2.5-mm NECT and we used 1-mm NECT. Most studies included anterior circulation AIS patients, while Shu et al. (21) reported 51 cases of posterior circulation stroke; Jagani et al. (22) also included 19 (16%) cases.

**Table 3:** Baseline characteristics and main outcomes of included studies (quantitative synthesis).

**References**	**No. of patients**	**Treatment modalities and devices (*n*, %)**	**Method of ROI drawing**	**Age**	**Male *n*, %)**	**NIHSS**	**IVT administered (*n*, %)**	**Stroke etiology (*n*, %)**	**Thrombus location (*n*, %)**	**Thickness (mm)**	**Successful reperfusion mean ± SD HU (*n*)**		
											**Yes**	**No**	***P*-value**
Yilmaz et al. (18)	70	SR (Solitaire FR, pREset, Trevo, Aperio)	Manually drawn within thrombus	72 ± 12	37 (52.9%)	NA	36 (51.4%)	NA	M1	5	50.1 ± 7.8 (57)	48.7 ± 8.2 (13)	0.567
Spiotta et al. (19)	95	Routinely penumbra; first-generation SR available	Manually drawn small circles	NA	NA	NA	32 (33.7%)	NA	NA	4.8	57.1 ± 16.3 (72)	68.7 ± 43.2 (23)	0.22
Mokin et al. (20)	41	SR (Solitaire)	Manually outlined the margin	67.9 ± 12.869.1 ± 12.5	12 (50%) 5 (29.4%)	17.0 ± 4.216.4 ± 4.3	8 (33.3%) 5 (29.4%)	NA	ICA terminus; M1; M2	5	49.9 ± 7.6 (24)	43.8 ± 6.6 (17)	0.01
Shu et al. (21)	51	Aspiration (13, 25.5%); SR (27, 52.9%); Aspiration and SR (2, 3.9%); Other (9, 17.6%)	Manually outlined the margin	72 (66–68)	36 (70.6%)	18 (14–23)	8 (15.7%)	LAA (30, 58.8%); CE (13, 25.5%); Other (5, 9.8%); Unknown (3, 5.9%)	Proximal BA (10, 19.6%); Mid BA (21, 41.2%); Tip BA (20, 39.2%)	2.5	52.3 ± 5.8 (41)	48.4 ± 6.7 (10)	0.07
Jagani et al. (22)	118	Aspiration (30, 25%); SR (30, 25%); Aspiration and SR (25, 21%); Other (33, 28%)	Manually drawn within thrombus	66 ± 13	62 (53%)	NA	NA	LAA (35, 30%); CE (56, 47%); Other (6, 5%); Unknown (21, 18%)	MCA/M1 (65, 55.1%); Intracranial ICA (5, 4.2%); T Occlusion (13, 11.0%); L Occlusion (16, 13.5%); BA (19, 16.1%)	5	50.1 ± 7.4 (80)	53.0 ± 12.7 (38)	0.17
Ye et al., 2019 (the present study)	64	Aspiration (26, 40.6%); SR (21, 32.8%); Aspiration and SR (17, 26.6%);	Manually drawn within thrombus	74.5 (63.25-82)	34 (53.1%)	14.5 (11–19)	19 (29.7%)	LAA (16, 25.0%); CE (37, 57.8%); Other (2, 3.1%); Unknown (9, 14.1%)	ICA (18, 28.1%); M1 (25, 39.1%); Tandem 7 (10.9%) Distal 14 (21.9%)	1	65.27 ± 9.42 (57)	62.19 ± 17.01 (7)	0.462
	**References**	**No. of patients**	**Treatment strategies**	**Thickness (mm)**	**Method of ROI drawing**	**Age**	**Male (*n*, %)**	**NIHSS**	**IVT administered (*n*, %)**	**Stroke etiology (*n*, %)**	**Thrombus location (*n*, %)**	**Statistical method**	**Conclusion**
Original article	Froehler et al. (23)	67	Merci	4.8	Manually drawn small circles or drawn within thrombus	69.3	26 (38.8%)	19	23 (34.3%)	NA	ICA; M1	Chi-square or Fisher's exact test	Successful recanalization was achieved in 79% (33/42) of patients exhibiting the HVS compared with only 36% (9/25) of patients without the HVS.
	Moftakhar et al. (24)	90	MT (Merci or Penumbra)	2.5	Manually drawn within thrombus	69	43 (47.8%)	NA	NA	NA	NA	Student *t* tests	Patients with TICI ≥ 2 demonstrated higher HU on NECT (mean corrected HU mechanical treatment = 1.7) compared with patients with TICI <2 (mechanical treatment = 1.3) (*P* = <0.0001).
	Kim et al. (25)	212	SR Aspiration and SR Other	4.8	NA	72 (65–79)	107 (50.5%)	13 (10–16)	143 (67.5%)	LAA (126, 59.4%); CE (45, 21.2%); Unknown (41, 19.3%)	M1; M2	Chi-square or Fisher's exact test	There were no significant differences between patients with an HMCAS and those with a negative HMCAS in the rates of successful reperfusion (83.1 vs. 81.9%, *P* = 0.829).
Conference abstract	Cardona et al. (26)	183	SR	NA	NA	64.5 ± 12	99 (54%)	17 (6–26)	97 (53.0%)	NA	M1 (142, 77.6%); M2 (25, 13.7%); T-ICA (16, 8.7%)	ROC analysis	In receiver operating characteristic (ROC) analysis attenuations of the clots was associated with a higher rate of recanalization TICI 2b-3 (AUC = 0.782;numbers of tumor-infiltrating *p* = 0.001).
	Kenmuir et al. (27)	408	Manual aspiration; or SR mediated manual aspiration	NA	NA	67.7	216 (53%)	17	NA	NA	MCA; BA	Chi-square or Fisher's exact test	TICI 2b/3 reperfusion on first-pass was associated with the absence of HDVS (*p* = 0.001).

*Data were represented as mean ± SD or median (IQR)*.

*Mokin et al. (20) reported age, the proportion of sex, and NIHSS according to successful reperfusion or not (upper-successful reperfusion; below-unsuccessful reperfusion)*.

*ROI, region of interest; NIHSS, National Institutes of Health Stroke Scale; IVT, intravenous thrombolysis; HU, Hounsfield unit; LAA, large artery atherosclerosis; CE, cardiogenic embolism; ICA, internal carotid artery; MCA, middle cerebral artery; BA, basilar artery; PCA, posterior cerebral artery; SR, stent retriever; ROC, receiver operating characteristic; HVS/HDVS, hyperdense vessel sign; MT, mechanical thrombectomy; TICI, thrombolysis in cerebral ischemia; HMCAS, hyperdense middle cerebral artery sign; NA, not available*.

#### Qualitative Assessment of HU Value for Reperfusion Outcomes

A total of 11 studies were included in systematic review reporting the association between thrombus density and reperfusion outcomes of thrombectomy (Table 3). The results of qualitative assessment were summarized in Table 4. Four studies suggested the correlation between higher thrombus density and successful reperfusion, and all these four studies used SR as the main thrombectomy strategy. Six studies found that there was no association between thrombus density and reperfusion outcomes, among which four adopted multiple treatment strategies. Only one study observed that lower density thrombus was prone to recanalization, which was mainly based on the CA technique.

**Table 4:** Qualitative assessment of HU value for reperfusion.

**Primary strategy**	**Number of studies**	**Favor higher HU in successful reperfusion group**	**Neutrality**	**Favor lower HU in successful reperfusion group**
All included studies	11	4	6	1
Primary SR	5	4	1	0
Primary CA	2	0	1	1
Multiple treatment strategies	4	0	4	0

*SR, stent retriever; CA, contact aspiration; NA, not available*.

#### Quantitative Synthesis of HU Value for Reperfusion Outcomes

**Figure 3:**
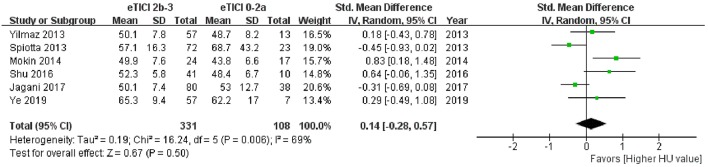
Forest plot shows the pooled results of meta-analysis.

Among the 11 studies, 6 articles had data available for meta-analysis. The results of meta-analysis and the forest plot were shown in Table 5 and Figure 3. The pooled results showed a comparable HU value of thrombus between eTICI2b-3 and eTICI0-2a reperfusion (SMD 0.14, 95%CI −0.28 to 0.57, *p* = 0.50) with moderate heterogeneity between studies (*p* = 0.006, *I*^2^ = 69%). Sensitivity analysis indicated that there was no study significantly affecting the pooled results, but significant publication bias was observed according to the Egger test (*p* = 0.046) (Supplementary Table 1).

**Table 5:** Pooled and subgroup outcomes.

	**Number of studies**	**SMD (95%CI)**	***P*-value**	***I*^**2**^**	**Subgroup differences**
All included studies	6	0.14 (−0.28 to 0.57)	0.50	69%	
Subgroups analysis					*p* = 0.007
Primary SR	3	0.53 (0.10 to 0.96)	0.02	14%	
Primary CA	2	−0.48 (−0.88 to −0.07)	0.02	0%	
Primary Solumbra	1	0.48 (−0.58 to 1.54)	0.37	NA	
Multiple treatment strategies	3	0.15 (−0.47 to 0.77)	0.64	67%	

*SR, stent retriever; CA, contact aspiration; Solumbra, a combination of SR and aspiration; SMD, standardized mean difference; CI, confidence interval; NA, not available*.

**Figure 4:**
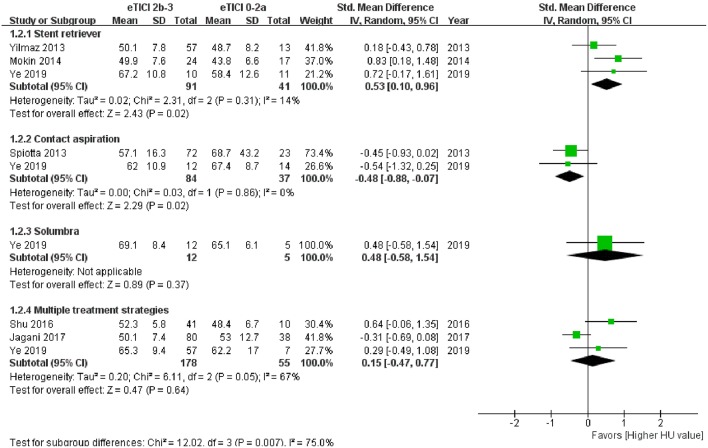
Forest plot shows subgroup analysis of meta-analysis.

Subgroup analysis of meta-analysis and its forest plot were displayed in Table 5 and Figure 4. For the SR subgroup, a significantly higher thrombus density was shown in eTICI2b-3 reperfusion compared with eTICI0-2a reperfusion group (SMD 0.53, 95%CI 0.10–0.96, *p* = 0.02) with mild heterogeneity (*p* = 0.31, *I*^2^ = 14%), while a significant inverse tendency was observed in the CA subgroup (SMD −0.48, 95%CI −0.88 to −0.07, *p* = 0.02) with no heterogeneity (*p* = 0.86, *I*^2^ = 0%). Only our single-center study had data available for primary Solumbra subgroup, and a similar HU value of thrombus was observed between eTICI2b-3 and eTICI0-2a reperfusion (SMD 0.48, 95%CI −0.58 to 1.54, *p* = 0.37). As for multiple treatment modalities subgroup, a comparable thrombus density was also showed between eTICI2b-3 and eTICI0-2a reperfusion (SMD 0.15, 95%CI −0.47 to 0.77, *p* = 0.64) with moderate heterogeneity (*p* = 0.05, *I*^2^ = 67%).

## Discussion

Both our single-center study and the pooled result of meta-analysis did not show an association between thrombus density and reperfusion outcomes of MT. Interestingly, subgroup analysis of the SR technique showed that thrombus density in the successful reperfusion group was significantly higher than that in the unsuccessful reperfusion group. At the same time, an inverse correlation was observed for the CA technique suggesting an association between lower thrombus density and successful reperfusion. This finding implicates a complementary role of SR and CA technique for different thrombus density and might be helpful for individualized thrombectomy strategy.

The ASTER trial and COMPASS trial suggested that the first-line CA technique was not inferior to the first-line SR for AIS patients (28, 29). By analyzing the cases of ASTER trial, Bourcier et al. further found that first-line SR could achieve higher reperfusion and better clinical outcome than first-line CA for patients with susceptibility vessel sign (SVS) on magnetic resonance imaging (MRI), which might help optimize the thrombectomy strategy (30). Apart from multimodal MRI, multimodal CT is also widely used for the preoperative evaluation of AIS patients. However, there is still a lack of corresponding research, based on pre-interventional CT imaging. As a consequence, we made a single-center study, yet no positive result was achieved, which may be limited by the study sample size. Then, we further updated the meta-analysis on this issue and the subgroup analysis indicated that SR and CA might have different efficacy for thrombus with a different HU value.

In the systematic review, five studies adopted SR as the main treatment strategy, among which four studies suggested a correlation between higher thrombus density and successful reperfusion (18, 20, 23, 24, 26). Subgroup analysis of meta-analysis for the SR technique also showed a significantly higher thrombus density in the successful reperfusion group. As higher thrombus density indicated higher erythrocyte content (9–11), the efficacy of SR for high-density thrombus could also be verified by several histologic studies. After histopathologic analysis for 83 cases of thrombus, Hashimoto et al. found that erythrocyte content (>64%) was positively related to successful reperfusion (7). Furthermore, by analyzing 79 AIS patients, Maekawa et al. suggested that RBC-rich thrombus needed less reperfusion time and fewer number of passes to retrieve compared with fibrin-rich clot (9). Recently, Duffy et al. conducted histologic analysis for thrombus, retrieved by each pass, and suggested that the content of thrombus fragments retrieved in passes 1 and 2 was significantly higher than that retrieved in passes 3–6 (31). Notably, all the histologic studies mainly used SR strategy, which further indicated that RBC-rich clot might be easier to retrieve through SR. These findings were also in line with the *post-hoc* analysis of ASTER trial to some extent (30) because a higher HU value on NECT and SVS on MRI suggested a similar histologic feature of thrombus (RBC-rich clot) (10). Therefore, the RBC-rich nature could be the underlying mechanism for the association between higher thrombus density and SR reperfusion, because this kind of thrombus was usually fresh (soft) and prone to integrate with stent (32). In contrast, the fibrin-rich clot was usually organized (hard) and might have a higher friction coefficient, so it was refractory to retrieve through SR (32, 33).

Among the included studies, only two (one conference abstract and one article) were focused on the CA technique (19, 27). After reviewing 408 cases, the conference abstract suggested that thrombus without hyperdense vessel sign was significantly associated with higher and faster reperfusion on first pass (27). This conclusion indicated that low-density thrombus might be prone to retrieve through manual aspiration. Another article also showed a relatively lower thrombus density in a successful reperfusion group compared with an unsuccessful reperfusion group (19). Moreover, the subgroup analysis of our meta-analysis showed a significant correlation between lower thrombus density and CA reperfusion (Figure 4). Based on these results, it seemed that the CA technique might be more efficient for lower density thrombus. As discussed, the lower density thrombus could indicate a fibrin-rich clot, which was relatively difficult to remove by SR. However, histologic study on the correlation between fibrin-rich clot and CA reperfusion was still limited and controversial. Maekawa et al. suggested that some fibrin-rich thrombi failed to retrieve using SR and could be successfully removed through the CA technique (9). Jagani et al. also indicated that the reperfusion rate of the CA technique was relatively higher for low-density thrombus (<50 HU) (15/17, 88%) compared with high-density thrombus (>50 HU) (8/13, 62%). A different result was shown in the recent study on per-pass analysis of thrombus composition, which suggested that thrombus retrieved by CA has higher RBC content compared with SR (31). Further studies are needed to detect the relationship between thrombus density and CA reperfusion, as well as the histological mechanism.

The studies only using SR or CA strategy were published in the early years (18–20). With the advancement of the thrombectomy technique, combined techniques were also frequently used such as Solumbra, CAPTIVE, and SAVE (21, 22), yet few studies reported the association between thrombus density and reperfusion outcome of combined techniques. Comparing reperfusion rates by clot density, Jagani et al. found that the combined technique could achieve a relatively higher reperfusion rate for high-density (>50 HU) thrombus (11/12, 92%) compared with low-density (<50 HU) clot (9/13, 69%) (22). This result was in accord with our single-center study, indicating a relatively higher thrombus density in the Solumbra reperfusion group. Though there is no significant difference, both studies implicated that the combined technique might be prone to retrieve higher thrombus density, which was similar to a stent retriever.

Among the systematic review, all the four studies based on multiple treatment modalities (including SR, CA, combined technique, and others) found no association between thrombus density and reperfusion outcomes (21, 22, 25). On the one hand, when first-line SR or CA could not achieve successful reperfusion, combined techniques or rescued stent implantation might help recanalize refractory clots. As most occlusion vessel could achieve successful reperfusion in the end, the difference of the HU value between successful and unsuccessful reperfusion group could reduce. On the other hand, it might also be attributed to the complementary efficacy of SR and CA technique on different thrombus density as discussed above, which might also reduce the difference in thrombus density between the successful and unsuccessful reperfusion groups.

Several issues should be noted in our observational study. Most studies previously focused on the successful reperfusion (eTICI2b-3), while two meta-analyses suggested that the clinical outcome of eTICI2c-3 could be better than eTICI2b (34, 35). Additionally, there is increasing concern about first-pass reperfusion outcome because of its better clinical outcomes (36–38). As a result, the successful or complete reperfusion outcomes after first pass, primary strategy, and final outcome were all compared in our observational study. Similar to the subgroup analysis of meta-analysis, the complementary role between SR and CA technique could also be observed for all these main outcomes in our single-center study (Figure 4). Notably, the reperfusion outcomes after primary strategies of our single-center study were included in the subgroup analysis of meta-analysis instead of final reperfusion outcomes. The reperfusion outcomes after primary strategies of our study might be closer to the results of studies in early years (18–20), as rescue therapy might be rarely used at that time. Finally, as for thickness of NECT, we attempted to measure thrombus density on both 1- and 5-mm NECT at first, but we found that 5 mm is too thick to scan the center of the thrombus in many cases. As a result, the thrombus density was measured on 1-mm reconstructed NECT, which could be more accurate compared with 5-mm reconstructed NECT.

### Limitations

Our meta-analysis has several limitations. First, no randomized controlled trial and only six retrospective studies were included, so the selection and publication bias cannot be avoided. Next, CT scanners, parameters (including the slice thickness of NECT), and the measurement method for thrombus density could differ among studies, which could lead to a difference in the HU value of thrombus measured among included studies. Yet, this kind of influence could decrease, when the SMD was calculated in the meta-analysis. Third, some important baseline characteristics were missing in the included studies, such as the lesion location, thrombus length or clot burden score, stroke etiology, the proportion of tandem lesion, or intracranial atherosclerotic stenosis related occlusion. These factors might also be important confounding factors for successful reperfusion, which could result in heterogeneity among studies and reduce comparability between included studies. As for our observational study, the sample size was limited and retrospective nature could also result in selection bias; the baseline characteristics were also imbalance among the three groups, such as diabetes mellitus and thrombus location. In summary, conclusions should be drawn with caution and further studies are needed to verify these results.

## Conclusion

Taken together, both our observational study and the pooled result of meta-analysis show a comparable thrombus density between successful and unsuccessful reperfusion groups. Interestingly, subgroup analysis of meta-analysis for primary SR strategy suggested that thrombus density in the successful reperfusion group was significantly higher than that in the unsuccessful reperfusion group; while subgroup analysis for the CA technique showed an inverse tendency. Meanwhile, though there was no significant difference, these similar trends were also shown in our single-center study. These results indicated that the SR and CA technique might have different efficacies for thrombus with different density on preoperative NECT. This finding may be an implication for optimization of individualized thrombectomy strategy; however, further studies of high quality are needed.

## Data Availability

All data are available in the manuscript and/or the Supplementary Files.

## Ethics Statement

This is a retrospective observational study without informed consent. Ethics approval was obtained in ethics committee of Beijing Hospital.

## Author Contributions

GY designed the study, collected clinical data, measured the HU value of thrombus, performed the literature search and selection, extracted and analyzed the data, and wrote and revised the manuscript. RC collected the clinical data, measured the HU value of thrombus, performed the literature search and selection, extracted and analyzed the data, and participated in writing the manuscript. JL collected the clinical data, participated in the literature search and selection, and revised the manuscript. PQ participated in the data analysis and revised the manuscript. JC designed the study, monitored the measurement of thrombus density and data extraction, and revised the manuscript. DW designed the study, monitored the studies inclusion and data analysis, and revised the manuscript. He is the guarantor.

### Conflict of Interest Statement

The authors declare that the research was conducted in the absence of any commercial or financial relationships that could be construed as a potential conflict of interest.
