# Supplementary material for: Association Between Thrombus Density and Reperfusion Outcomes Using Different Thrombectomy Strategies: A Single-Center Study and Meta-Analysis
Source: Front Neurol. 2019 Aug 16;10:843. doi: 10.3389/fneur.2019.00843 (PMC6706902; doi:10.3389/fneur.2019.00843)
Supplement: Supplementary file 1 [file Table_1.DOCX]

**SUPPLEMENTARY**

**Risk of bias assessment in each study**

| **Year** | **Author** | **Selction (4)** | **Comparability (2)** | **Exposure (3)** |
| --- | --- | --- | --- | --- |
| 2013 | Yilmaz | ★★★ |  | ★★★ |
| 2013 | Spiotta | ★★★ |  | ★★ |
| 2014 | Mokin | ★★★ | ★ | ★★★ |
| 2016 | Shu | ★★★ | ★★ | ★★★ |
| 2017 | Jagani | ★★★ |  | ★★ |
| 2019 | Ye | ★★★ |  | ★★★ |

Note: Risk of bias was assessed by the modified Newcastle-Ottawa Scale.

**Mean difference of HU according to recanalization**

1. **Funnel plot**

**
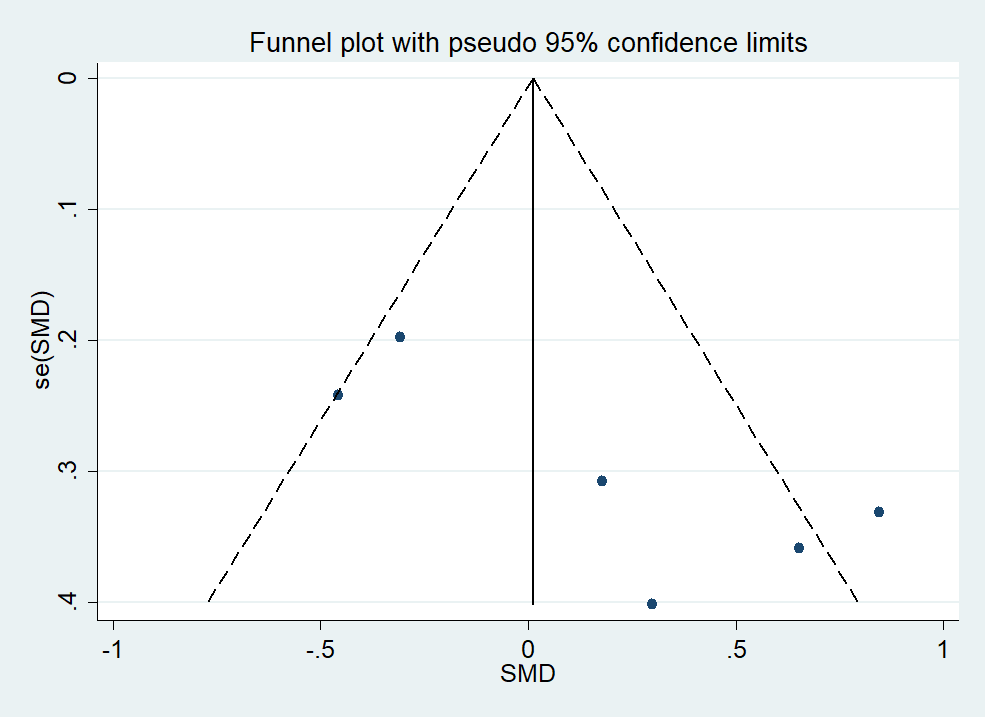
**

1. **Egger test: P= 0.046**

1. **Sensitivity analysis**
